# Long-term outcome of patients presenting with myocardial injury or myocardial infarction

**DOI:** 10.1007/s00392-023-02334-w

**Published:** 2023-11-20

**Authors:** Paul M. Haller, Caroline Kellner, Nils A. Sörensen, Jonas Lehmacher, Betül Toprak, Alina Schock, Tau S. Hartikainen, Raphael Twerenbold, Tanja Zeller, Dirk Westermann, Johannes T. Neumann

**Affiliations:** 1 Department of Cardiology, University Heart and Vascular Center Hamburg, Center for Population Health Innovation (POINT), University Medical Center Hamburg-Eppendorf, Building O50, Empore, Martinistrasse 52, 20246 Hamburg, Germany; 2 German Center for Cardiovascular Research (DZHK), Partner Site Hamburg/Kiel/Lübeck, Hamburg, Germany; 3 Department of Cardiology, University Heart Center Freiburg Bad Krotzingen, Bad Krotzingen, Germany; 4 University Center of Cardiovascular Science, University Heart and Vascular Center Hamburg, Hamburg, Germany; 5 Department of Epidemiology and Preventive Medicine, School of Public Health and Preventive Medicine, Monash University, Melbourne, Australia

**Keywords:** High-sensitivity cardiac troponin, Myocardial infarction, Myocardial injury, Long-term outcome, Fourth UDMI, Acute coronary syndrome

## Abstract

**Aims:**

Patients with acute or chronic myocardial injury are frequently identified in the context of suspected myocardial infarction (MI). We aimed to investigate their long-term follow-up.

**Methods and results:**

We prospectively enrolled 2714 patients with suspected MI and followed them for all-cause mortality and a composite cardiovascular endpoint (CVE; cardiovascular death, MI, unplanned revascularization) for a median of 5.1 years. Final diagnoses were adjudicated by two cardiologists according to the Fourth Universal Definition of MI, including 143 (5.3%) ST-elevation MI, 236 (8.7%) non-ST-elevation MI (NSTEMI) Type 1 (T1), 128 (4.7%) NSTEMI T2, 86 (3.2%) acute and 677 (24.9%) with chronic myocardial injury, and 1444 (53.2%) with other reasons for chest pain (reference). Crude event rates per 1000 patient-years for all-cause mortality were highest in patients with myocardial injury (81.6 [71.7, 92.3]), and any type of MI (55.9 [46.3, 66.7]), compared to reference (12.2 [9.8, 15.1]). Upon adjustment, all diagnoses were significantly associated with all-cause mortality. Moreover, patients with acute (adj-HR 1.92 [1.08, 3.43]) or chronic (adj-HR 1.59 [1.16, 2.18]) myocardial injury, and patients with NSTEMI T1 (adj-HR 2.62 [1.85, 3.69]) and ST-elevation MI (adj-HR 3.66 [2.41, 5.57]) were at increased risk for cardiovascular events.

**Conclusion:**

Patients with myocardial injury are at a similar increased risk for death and cardiovascular events compared to patients with acute MI. Further studies need to determine appropriate management strategies for patients with myocardial injury.

**Registration:**

Clinicaltrials.gov (NCT02355457).

**Supplementary Information:**

The online version contains supplementary material available at 10.1007/s00392-023-02334-w.

## Introduction

Patients with symptoms suggestive of myocardial infarction (MI) denote a considerable part of all patients presenting to the emergency department [1]. Of these, approximately one in five patients is diagnosed as having acute MI [2]. In recent years, fast diagnostic protocols based on the evaluation of two cardiac troponin values and their dynamic pattern using high-sensitive assays (hs-cTn) have improved the diagnostic management considerably [3, 4]. The ability to determine very small concentrations of cardiac troponin results in the early identification or rule out of MI, respectively [3–6]. The increased sensitivity also results in the frequent identification of patients with hs-cTn elevation due to other reasons apart from MI [2, 3, 7]. Among others, patients with chronic myocardial injury, defined as a stable elevation of troponin above the 99th percentile, are frequently identified [8].

Recent recommendations on the classification of patients by the Fourth Universal Definition of MI (UDMI) [7] recommend the differentiation of type 1 (T1) and type 2 (T2) MI. This classification differentiates a vessel thrombosis due to rupture or erosion of an atherosclerotic plaque, from (atherosclerotic-independent) myocardial oxygen supply and demand mismatch due to other reasons. In addition, the Fourth UDMI emphasized the groups of acute and chronic myocardial injury, describing patients without evident myocardial ischemia, but dynamic or stable hs-cTn elevation above the 99th percentile, respectively [7].

Previous analyses reported similar, or even higher event rates for patients with T2MI compared to patients with T1MI [2, 9]. However, prior studies were limited in the length of follow-up, reported diagnoses that have not been adjudicated independently, or did not compare all types of diagnoses according to the Fourth UDMI [8, 10, 11].

In addition, event rates for patients with myocardial injury are less clear if stratified according to acute and chronic injury [9]. Particularly these groups are usually composed of pre-diseased patients with multiple comorbidities and distinct management recommendations are scarce [2, 4]. Yet, if these patients were at an increased risk for cardiovascular events, the presentation to the emergency department with an identification of elevated troponin could act as a trigger for the introduction of specific interventions and preventive strategies, targeting at a reduction of cardiovascular risk and hereby modifying outcomes. Therefore, we aimed to study the prevalence of T1MI, T2MI, and acute and chronic myocardial injury, and determine the event rates for all-cause mortality, and a composite cardiovascular endpoint during long-term follow-up.

## Methods

### Study populations

The Biomarkers in Acute Cardiac Care (BACC) study is an ongoing, single-center observational cohort study prospectively enrolling patients with symptoms suggestive of acute MI and presenting to the emergency department of the University Medical Center Hamburg-Eppendorf. The study is registered with clinicaltrials.gov (NCT02355457) and has been described previously [2, 12–14]. All patients provided written informed consent; the study is conducted according to the Declaration of Helsinki and was approved by the appropriate ethics committees. Enrollment of patients started in July 2013. For the present secondary analysis, all patients enrolled from inception of the study up to July 2019 are considered (Supplementary Fig. 1).

### Clinical management and study procedures

Patients were managed by the physician in charge in the emergency department. Management followed institutional standards and respective guidelines as applicable [4, 15]. As a routine, patients received recordings of an electrocardiogram, routine laboratory diagnostics and imaging, all at the discretion of the physician in charge. Routinely, hs-cTnT (Elecsys, Roche Diagnostics, Basel, Switzerland) was determined at presentation (0 h) and after 1 and 3 h (1 h, 3 h) and used to guide the diagnostic and therapeutic management of patients. Study-specific procedures included biobanking of serum and plasma samples at 0 h, 1 h, and 3 h.

### Adjudication of the final diagnosis

All patients were adjudicated based on the results of hs-cTnT (Elecsys, Roche Diagnostics, Basel, Switzerland) by two independent cardiologists using all available information, with disagreement resolved by consultation of a third cardiologist. Adjudication was performed according to the Fourth Universal Definition of MI [7].

### Follow-up of patients

Patients were followed up to 8 years on a census-based system. Trained study personnel contacted all patients or their relatives by telephone to conduct a structured interview on events of interest, including all-cause mortality, cardiovascular death, acute MI, unplanned revascularization, cardiac re-hospitalization, and cardiac symptoms. In addition, hospital and ambulatory care reports were retrieved, and relevant information was extracted by a cardiologist. If patients or their relatives were unavailable multiple times, they were contacted by mail and E-mail, followed by contacting the family physicians. If no information was available after multiple attempts, the local death registry was consulted to retrieve information on a potential death, including date of death.

### Statistical analyses

Continuous variables are shown as median and interquartile range, and categorical variables as number and percentages. Our primary outcome of interest was all-cause mortality during follow-up. Secondary endpoint was a composite cardiovascular endpoint including cardiovascular death, incidental MI, or unplanned coronary revascularization. Crude event rates were provided per 1000 person-years with 95% confidence intervals (CI), calculated by Poisson Regression. The median follow-up time was estimated by the reverse Kaplan–Meier estimator [16]. Survival curves were estimated by the Kaplan–Meier method and groups were compared using log-rank test. Analyses were performed for the individual endpoints using Cox-proportional hazard models adjusted for age, sex, diabetes, smoking, hypertension, hyperlipoproteinemia, a family history of coronary artery disease (CAD), history of stroke, known congestive heart failure, and chronic kidney disease (defined as estimated glomerular filtration rate [eGFR] < 60 ml/min). Concentrations of hs-TnT are shown for each time point stratified by final diagnosis. Summarizing lines were computed for smoothed conditional means using local polynomial regression fitting (loess). All statistical analyses were performed using R version 4.1.2 (R Foundation for Statistical Computing) [17].

## Results

### Descriptive summary of the study population

The overall study population included 2714 patients. Of these, 143 (5.3%) had ST-elevation MI, 236 (8.7%) type 1 non-ST-elevation MI (T1MI), 128 (4.7%) type 2 non-ST-elevation MI (T2MI), 86 (3.2%) acute myocardial injury, 677 (24.9%) chronic myocardial injury, and the remaining 1444 (53.2%) patients had diagnoses without any hs-cTn elevation (Table 1). The latter constituted 206 (7.6%) patients with unstable and 18 (0.7%) with stable angina pectoris (AP), respectively, as well as 396 (14.6%) patients with cardiac non-coronary chest pain, and 824 (30.4%) patients with non-cardiac chest pain. The median follow-up was 5.1 (4.9, 5.2) years.

**Table 1:** Baseline characteristics

	All (*N* = 2714)	STEMI (*N* = 143)	NSTEMI 1 (*N* = 236)	NSTEMI 2 (*N* = 128)	Acute myocardial injury (*N* = 86)	Chronic myocardial injury (*N* = 677)	Others (*N* = 1444)
Age (years)	64.0 (51.0, 75.0)	65.0 (52.0, 72.0)	69.5 (60.0, 77.0)	71.5 (59.0, 78.0)	70.5 (59.2, 77.8)	75.0 (68.0, 81.0)	56.0 (46.0, 68.0)
Male no. (%)	1745 (64.3)	111 (77.6)	179 (75.8)	76 (59.4)	50 (58.1)	368 (54.4)	961 (66.6)
Body mass index (kg/m^2^)	26.1 (23.7, 29.7)	26.2 (23.8, 29.7)	26.6 (24.3, 29.6)	26.9 (24.1, 29.4)	25.7 (23.3, 30.8)	26.1 (23.5, 29.9)	26.0 (23.5, 29.4)
Hypertension no. (%)	1771 (65.4)	94 (65.7)	184 (78.3)	101 (78.9)	60 (69.8)	542 (80.3)	790 (54.7)
Hyperlipoproteinemia no. (%)	947 (34.9)	45 (31.5)	129 (54.7)	49 (38.3)	26 (30.2)	295 (43.6)	403 (27.9)
Diabetes no. (%)	342 (12.7)	16 (11.5)	53 (22.5)	14 (11.1)	14 (16.3)	135 (20.3)	110 (7.6)
Ever smoker no. (%)	1272 (47.5)	86 (61.0)	128 (54.7)	61 (48.0)	45 (52.9)	249 (37.6)	703 (48.7)
Family history of coronary artery disease no. (%)	510 (19.5)	33 (24.3)	62 (27.0)	19 (15.4)	6 (7.5)	71 (11.1)	319 (22.1)
Coronary artery disease no. (%)	898 (33.1)	30 (21.0)	115 (48.7)	48 (37.5)	34 (39.5)	321 (47.4)	350 (24.2)
Stroke no. (%)	163 (6.0)	4 (2.8)	16 (6.8)	7 (5.5)	8 (9.3)	61 (9.0)	67 (4.6)
Peripheral artery disease no. (%)	166 (6.1)	12 (8.4)	26 (11.0)	10 (7.8)	7 (8.1)	64 (9.5)	47 (3.3)
Chronic kidney disease (eGFR < 60 ml/min/1.73 m^2^) no. (%)	725 (26.9)	42 (29.6)	94 (39.8)	60 (47.2)	39 (46.4)	357 (53.2)	133 (9.2)
Congestive heart failure no. (%)	320 (11.8)	11 (7.7)	36 (15.3)	26 (20.3)	30 (34.9)	146 (21.6)	71 (4.9)
Atrial fibrillation no. (%)	402 (14.8)	8 (5.7)	18 (7.6)	48 (37.5)	22 (25.6)	192 (28.4)	114 (7.9)
LVEF normal	1733 (79.8)	27 (36.5)	132 (62.6)	67 (68.4)	35 (52.2)	355 (69.7)	1117 (77.4)
LVEF mildly/moderately reduced	325 (15.0)	35 (47.3)	62 (29.3)	23 (23.5)	16 (23.8)	106 (20.8)	83 (5.8)
LVEF severely reduced	114 (5.2)	12 (16.2)	17 (8.1)	8 (8.2)	16 (23.9)	48 (9.4)	13 (0.9)
eGFR (mL/min for 1.73 m^2^)	76.8 (58.5, 92.3)	74.8 (58.7, 91.4)	66.0 (48.8, 82.4)	61.4 (44.7, 82.3)	64.3 (43.1, 87.0)	58.3 (44.5, 76.7)	86.4 (71.8, 97.6)
hs-TnT 0 h (ng/L)	10.0 (5.0, 23.0)	70.0 (17.0, 460.0)	89.5 (31.8, 243.0)	23.0 (13.0, 59.2)	70.0 (29.0, 204.0)	19.0 (14.0, 30.0)	5.0 (3.0, 8.0)
hs-TnT absolute 1-h delta (ng/L)	1.0 (0, 3.0)	85.0 (38.0, 314.0)	15.0 (5.0, 34.0)	8.0 (4.0, 14.5)	10.0 (3.0, 25.0)	1.0 (1.0, 3.0)	1.0 (0, 1.1)
Angiography at index presentation no. (%)	803 (29.6)	142 (99.3)	221 (93.6)	56 (43.8)	39 (45.3)	167 (24.7)	178 (12.3)
Myocardial revascularization during index presentation no. (%)	456 (16.8)	132 (92.3)	178 (75.4)	6 (4.7)	3 (3.5)	76 (11.2)	61 (4.2)

Baseline characteristics are shown for the overall study population and stratified by the adjudicated final diagnoses. The last group “others” gathers patients with final diagnoses not involving any elevation of high-sensitivity cardiac troponin T, including stable angina pectoris, unstable angina pectoris, cardiac non-coronary chest pain, and non-cardiac chest pain. Numbers are shown as median (25th percentile, 75th percentile) and categorical variables as absolute number (percent). *eGFR* estimated glomerular filtration rate; *hs-cTnT* high-sensitivity cardiac troponin T; *LVEF* left ventricular ejection fraction

Patients with chronic myocardial injury were the oldest, more frequently female, and with the highest prevalence of hypertension and chronic kidney disease, and with known CAD in almost every second patient. Patients with acute myocardial injury had the highest prevalence of known congestive heart failure, valvular heart disease, and a history of stroke. Details on all patient characteristics by final diagnoses are provided in Table 1. Ambulatory medication documented at baseline, findings of myocardial stress tests for patients with T2MI, acute myocardial injury, and chronic myocardial injury, as well as the most likely cause of T2MI, acute myocardial injury, and chronic myocardial injury are provided in Supplementary Tables 2 and 3, respectively.

**Fig. 1:**
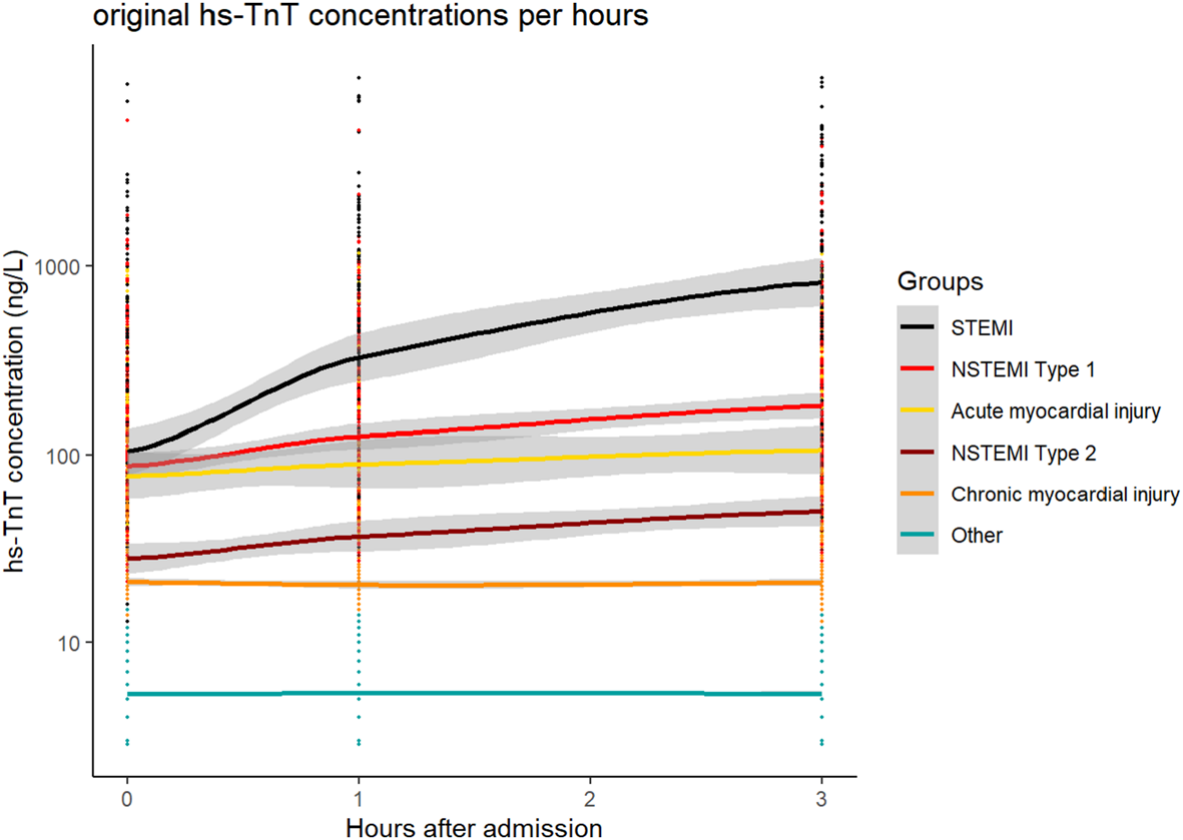
Course of high-sensitivity cardiac troponin T at baseline (0 h), after 1 h, and 3 h stratified by final diagnosis according to the Fourth Universal Definition of Myocardial Infarction. The y-axis shows the absolute concentration on a log-transformed scale. The group “others” gathers patients with final diagnoses not involving any elevation of high-sensitivity cardiac troponin T, including stable angina pectoris, unstable angina pectoris, cardiac non-coronary chest pain, and non-cardiac chest pain. NSTEMI—non-ST-elevation myocardial infarction; STEMI—ST-elevation myocardial infarction

The serial hs-cTnT concentrations stratified by final diagnoses at baseline and after 1 h and 3 h are shown in Fig. 1. Within all diagnoses with elevated hs-cTnT, patients with chronic myocardial injury had the lowest median values and the highest increase was observed in those with STEMI. Patients with acute myocardial injury had higher absolute hs-cTnT concentrations than patients with T2MI.

### Crude event rates in the study population

The crude event rate in the entire cohort for all-cause mortality was 37.8 [34.4, 41.4] events per 1000 person-years, and 34.7 [31.2, 38.4] events per 1000 person-years for the composite endpoint (Table 2).

**Table 2:** Event rates per 1000 patient-years

	Entire cohort	Myocardial injury	Myocardial infarction	All other patients
Death	37.78 (34.35, 41.44)	81.59 (71.72, 92.33)	55.91 (46.34, 66.71)	12.24 (9.75, 15.12)
Cardiovascular mortality, myocardial infarction, or unplanned revascularization	34.71 (31.23, 38.44)	56.34 (47.45, 66.27)	69.45 (57.57, 82.87)	16.62 (13.6, 20.06)

Event rates per 1000 patient-years (with 95% confidence interval) are provided for the complete follow-up for the entire study population. Further stratification has been performed for patients with myocardial injury (acute or chronic), myocardial infarction (ST-elevation myocardial infarction, non-ST-elevation myocardial infarction type 1, and non-ST-elevation myocardial infarction type 2), and all remaining patients without either diagnosis

The highest event rates for all-cause mortality were observed in patients with either form of myocardial injury (81.6 [71.7, 92.3] events per 1000 person-years), and in patients with any type of MI (55.9 [46.3, 66.7] events per 1000 person-years), compared to patients without hs-cTn elevations (12.2 [9.8, 15.1] events per 1000 person-years).

**Fig. 2:**
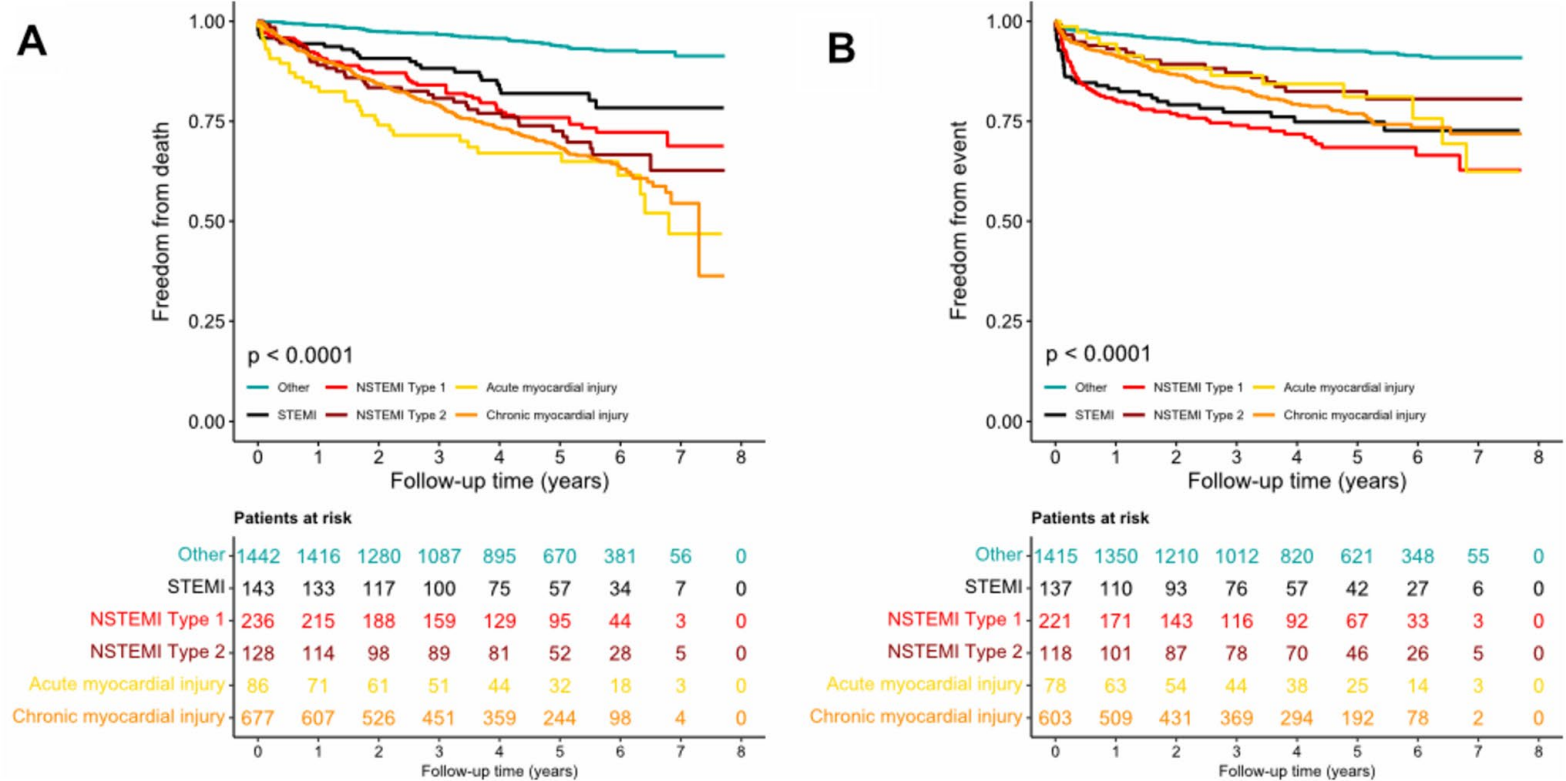
Kaplan–Meier plots are shown for the entire study population stratified by the final diagnoses for all-cause mortality (**A**) and a cardiovascular composite endpoint (**B**), including cardiovascular death, incidental acute myocardial infarction, or unplanned revascularization. Statistical comparison was performed using log-rank test. The group “others” gathers patients with final diagnoses not involving any elevation of high-sensitivity cardiac troponin T, including stable angina pectoris, unstable angina pectoris, cardiac non-coronary chest pain, and non-cardiac chest pain

Similarly, patients with MI (69.5 [57.6, 82.9] events per 1000 person-years) and myocardial injury (56.3 [47.5, 66.3] events per 1000 person-years) had the highest event rates compared to the patients without hs-cTn elevation (16.2 [13.6, 20.1] events per 1000 person-years) for the composite cardiovascular endpoint (Table 2). Figure 2 provides the Kaplan–Meier plot for either endpoint stratified by final diagnoses.

### Adjusted outcome analyses

**Fig. 3:**
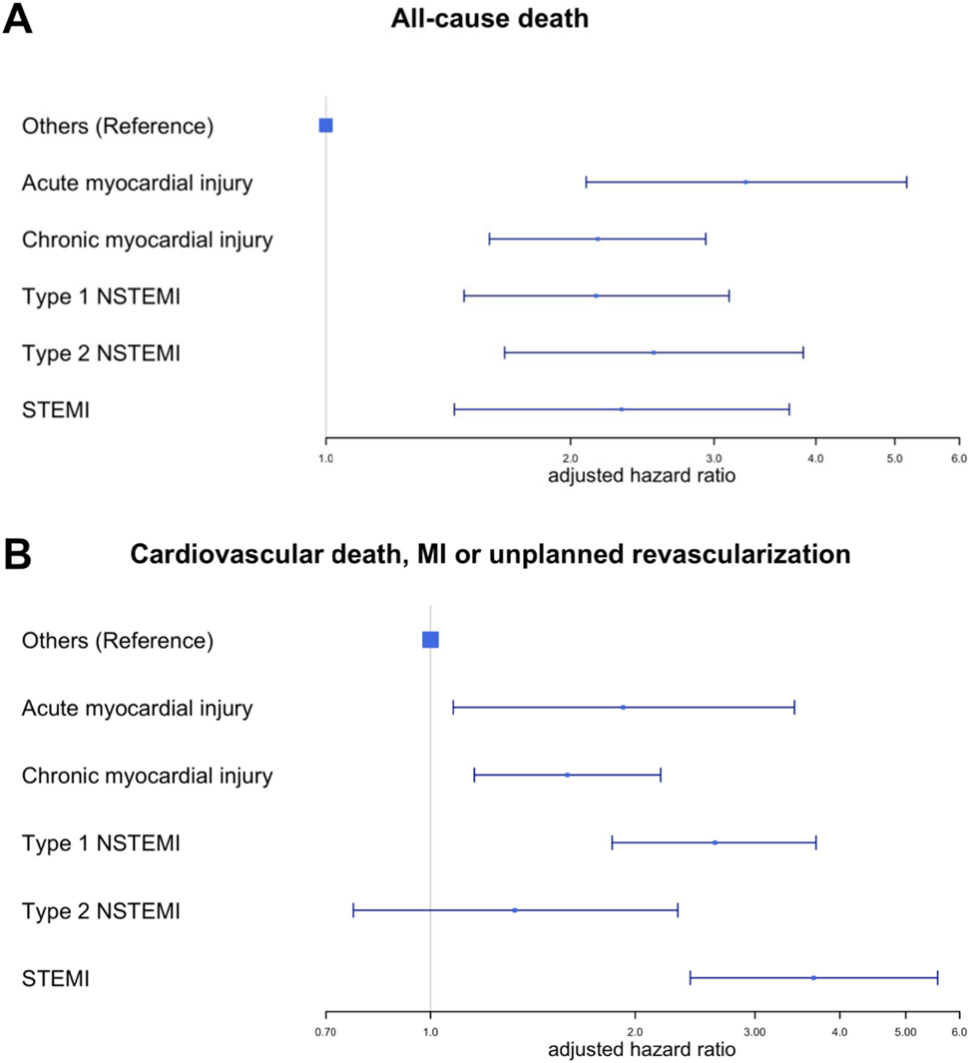
Results of Cox-regression analyses investigating the risk for all-cause mortality (**A**) or a cardiovascular composite endpoint (**B**) for the final diagnoses according to the Fourth Universal Definition of Myocardial Infarction during long-term follow-up. Adjustment was made for age, sex, diabetes, smoking, hypertension, hyperlipoproteinemia, a family history of coronary artery disease, history of stroke, known congestive heart failure, and chronic kidney disease (defined as estimated glomerular filtration rate [eGFR] < 60 ml/min). The group “others” gathers patients with final diagnoses not involving any elevation of high-sensitivity cardiac troponin T, including stable angina pectoris, unstable angina pectoris, cardiac non-coronary chest pain, and non-cardiac chest pain

Compared to patients without hs-cTnT elevation and upon adjustment for age, sex, cardiovascular risk factors, and a history of stroke, congestive heart failure, and chronic kidney disease, all patients with either type of MI or myocardial injury had a significantly higher risk for all-cause mortality during follow-up (Fig. 3A). The risk was comparable between all these groups using patients without hs-cTnT elevation and other causes of chest pain as a reference: acute myocardial injury (adj-HR 3.28 [2.09, 5.17], *p* < 0.001), chronic myocardial injury (adj-HR 2.16 [1.59, 2.93], *p* < 0.001), T1MI (adj-HR 2.15 [1.48, 3.13], *p* < 0.001), T2MI (adj-HR 2.53 [1.66, 3.86], *p* < 0.001), and STEMI (adj-HR 2.31 [1.44, 3.71], *p* < 0.001, Supplementary Table 4).

With respect to the composite cardiovascular endpoint including cardiovascular death, MI, or unplanned revascularization (Fig. 3B), we observed an increased risk for all groups but T2MI: acute myocardial injury (adj-HR 1.92 [1.08, 3.43], *p* = 0.027), chronic myocardial injury (adj-HR 1.59 [1.16, 2.18], *p* = 0.0037), T1MI (adj-HR 2.62 [1.85, 3.69],* p* < 0.001), T2MI (adj-HR 1.33 [0.77, 2.31], *p* = 0.3), and STEMI (adj-HR 3.66 [2.41, 5.57], *p* < 0.001), with further details presented in Supplementary Table 5.

### Myocardial injury and cardiovascular outcome

**Fig. 4:**
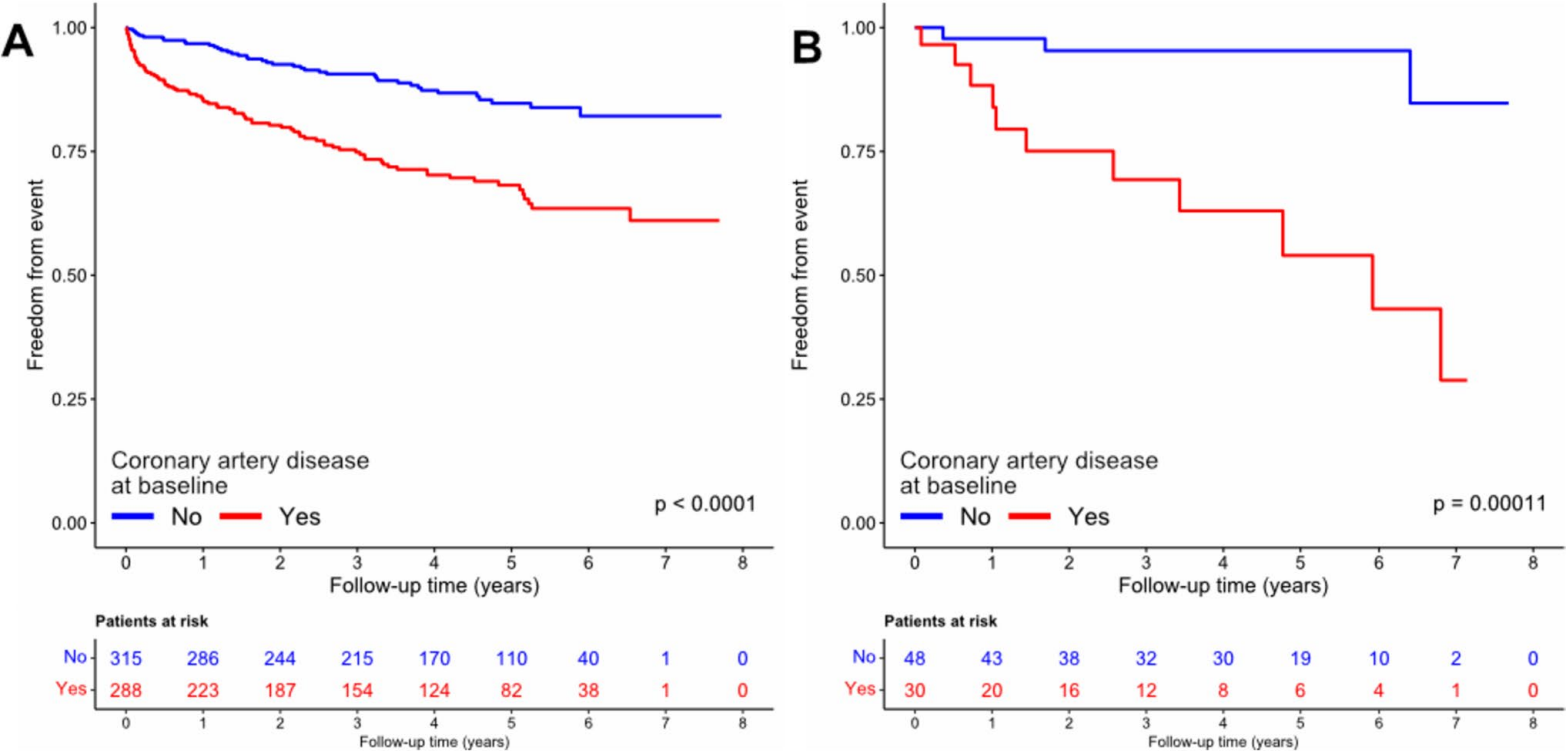
Kaplan–Meier plots are shown for the sub-groups of chronic myocardial injury (**A**) and acute myocardial injury (**B**) investigating a composite cardiovascular endpoint (including cardiovascular death, myocardial infarction, or unplanned revascularization). Patients are stratified by a history of coronary artery disease at baseline. Group comparison was performed using log-rank test

To further elucidate the high number of cardiovascular events in the patient groups of acute and chronic myocardial injury, respectively, we investigated whether prevalent CAD at baseline reasonably stratified patients with and without cardiovascular events during follow-up. Figure 4 provides the incidence for the composite cardiovascular endpoint for patients with acute (Fig. 4A) and chronic (Fig. 4B) myocardial injury, stratified by prevalent CAD. We observed significantly more events in those with CAD in both patient group (*p* = 0.00011 and *p* < 0.001, respectively).

## Discussion

In this manuscript, we report the cardiovascular risk of patients presenting with suspected acute MI during a median long-term follow-up of 5 years. As a key finding, we show that patients with myocardial injury are at comparable risk for death and cardiovascular events to those presenting with acute MI. The increased recognition of acute and chronic myocardial injury as separate entities apart from MI has important clinical implications, as has the differentiation of T1MI and T2MI. The ability to differentiate these different types of myocardial injury is partly owed to the increased sensitivity of cardiac troponin assays, hereby frequently revealing slightly elevated troponin concentrations [3]. Considering the increasing availability of hs-cTn assays, a rising number of clinicians is confronted with the interpretation of partly non-ischemic elevations of troponin. Understanding the prevalence of such myocardial injury, as well as their prognosis is of clinical importance to guide further treatment. The present study investigated the long-term outcome of patients admitted to the chest pain unit of a tertiary care center, with final diagnoses adjudicated according to the Fourth Universal Definition of MI [7]. Compared to earlier studies, our study is unique, as it is based on thorough state-of-the-art adjudication and has extended long-term follow-up available. The following observations have important implications for clinical routine:

First, myocardial injury is very common. Overall, 3.2% of patients have been classified with acute myocardial injury (almost comparable to the prevalence of T2MI in the present cohort) and even every fourth patient in the overall cohort had chronic myocardial injury. Thus, myocardial injury was more frequent than all types of MI combined.

Second, despite the absence of infarction at presentation, the crude event rate per 1,000 patient-years for all-cause mortality was highest in those with myocardial injury. As shown in Fig. 2A, the crude rate of all-cause mortality was highest in those with myocardial injury, with STEMI patients having the best survival of all patients with a diagnosis of MI or myocardial injury. The high risk for all-cause mortality remained highly significant for myocardial injury and infraction upon adjustment for age, sex, cardiovascular risk factors, and important clinical conditions. In fact, we did not observe any differences in risk comparing these groups of patients to those with MI.

Third, a previous study reported an equal rate of major adverse cardiovascular events comparing patients with T2MI or myocardial injury with T1MI patients [9]. In the present study, we can confirm a high risk for cardiovascular events in patients with T1MI, STEMI and those with myocardial injury. Yet, in the fully adjusted model, patients with T2MI were not at significantly increased risk for this composite cardiovascular endpoint in our study.

Compared to previous reports, we were able to differentiate acute and chronic myocardial injury, allowing a more specific evaluation of event rates for both types during long-term follow-up. Here we prove that the risk for cardiovascular death, MI or unplanned revascularization was also significantly increased in patients with acute or chronic myocardial injury, despite full adjustment for patient characteristics, cardiovascular risk factors, and underlying comorbidities. Although we observed the sharpest increase in the incidence of the composite cardiovascular endpoint (including cardiovascular death, MI, or unplanned revascularization) in patients with STEMI or T1MI, the risk seemed to decrease over time. If the entire follow-up is considered, the incidence remained largely similar with patients having either acute or chronic myocardial injury. In these groups, particularly those patients with known CAD experienced cardiovascular events, as shown in Fig. 4A and B.

Fourth, out of all diagnoses requiring a troponin elevation exceeding the 99th percentile, the median elevation in troponin concentrations was lowest in patients with chronic myocardial injury (median 19 ng/L, 99th percentile upper reference limit of the hs-cTnT troponin assay 14 ng/L). Also, it was the most frequent diagnosis involving troponin elevation. Irrespective of the comparably low troponin elevation, clinicians should be aware of the high risk for death and cardiovascular events at which these patients are. Identification of patients at higher risk should ideally result in the initiation of therapies aiming at a risk reduction. In the case of patients with myocardial injury, data on this matter are scarce. Yet, presentation to the emergency department and identification of chronic myocardial injury could act as a trigger for intensified primary or secondary preventive therapies. For instance, the prevalence of known CAD in patients with chronic myocardial injury was high. Almost every second patient had a history of CAD, that was associated with the highest risk for cardiovascular events (2.56 [1.97, 3.33], Supplementary Table 5). Identification of those patients should prompt physicians to assess secondary prevention therapies established (for example, lipid lowering therapy) and, ideally, increase those per guidelines to recommended targets and tolerated doses [18, 19]. Although dedicated trials for such an approach (identification of patients by elevated hs-cTn resulting into intensification of therapy) have not been conducted to date, lipid lowering therapy results in a strong risk reduction and sub-studies suggest  that hs-cTn concentrations decrease along with an intensification of lipid lowering therapy accompanied by a risk reduction [20, 21].

In addition to CAD, patients with chronic myocardial injury frequently presented with other important comorbidities as well, among others chronic kidney disease (53.2%), atrial fibrillation (28.4%), heart failure (21.6%), or severe valvular heart defects (17.5%). Thus, one may speculate whether a structured assessment in patients with chronic myocardial injury allows an improvement of therapy, which could translate into a decrease in hs-cTn, as also to a reduction in the high event rate observed. In this regard, previous studies could already demonstrate that a reduction in cardiac troponin parallels a reduction in the risk for cardiovascular events, and that specific therapeutic measures mitigate both [20–22].

Fifth, previous studies have reported a rather wide range with respect to the prevalence of T2MI [9, 23–25], which might be owed to different settings and patient samples enrolled. Yet, T2MI is very common and in our study, approximately one out of three patients with non-ST-elevation MI had T2MI. In line with previous reports [9], the risk for all-cause mortality is high for patients with T2MI, with even higher crude event rates compared to patients with T1MI or STEMI [9, 25]. While the risk for all-cause mortality remained significantly elevated upon adjustment for patient characteristics and comorbidities in these patients, the risk for the composite cardiovascular endpoint did not, an observation similarly to a report by Chapman et al. [9]. Overall, patients with T2MI were commonly older and presented with multiple cardiovascular risk factors and comorbidities, probably partly explaining the high observed event rate. However, since the risk for cardiovascular events in our model was reduced upon full adjustment for patients with T2MI, it may be hypothesized that most likely non-cardiovascular causes drive the high mortality rate observed in these patients. Considering the high prevalence of cardiovascular risk factors in this group of patients, as also the high prevalence of CAD, it may be speculated whether competing risk, with patients dying due to non-cardiovascular causes before cardiovascular events could happen, might also explain some of the observations in our study and others [9]. Irrespective of the multiple cardiovascular risk factors, the ambulatory therapy as provided in Supplementary Table 1 leaves room for appropriate secondary prevention therapies.

## Strengths and limitations

A major strength of our study is that it is based on a large, contemporary sample of well-characterized patients and who were followed for a very long period thereafter. However, a long period of enrollment could also result in a bias over time. Although enrollment takes place according to standardized approaches since implementation of the study, we cannot rule out to the full extent internal or external factors potentially affecting patient enrollment. Among others, this could apply to changes in patient presentation affecting the studied population (as an example of external factors), or changing study or chest pain unit staff, respectively, (as an example of internal factors). Yet, in internal analyses, we did not observe differences with respect to mortality of patients enrolled early or late in the trial (data not shown). The reported prevalence of each final diagnosis may differ from other cohort studies and may be influenced by the overall health care system and patient flow. The process of adjudication of the final diagnosis targets at an unbiased interpretation of all available data and is conducted by at least two independent cardiologists and a third cardiologist if needed. However, due to the nature of this process and the missing gold standard for diagnosing MI apart from troponin, there is room for potential misclassification.

## Conclusion

In conclusion, we show that myocardial injury is very common, and despite the absence of acute MI, these patients are at high risk for death and cardiovascular events. Thus, identification of myocardial injury should trigger careful diagnostic and therapeutic management, aiming at the implementation of appropriate primary and secondary prevention therapies. Also, patients with T2MI are at an increased risk for death, although event rates in these patients are most likely driven by non-cardiovascular causes.

## Supplementary Information

Below is the link to the electronic supplementary material.

Supplementary file1 (DOCX 97 KB)

## Data Availability

The data underlying this article cannot be shared publicly due to legal and institutional restrictions and policies. The data that support the findings of this study are available on reasonable request to the corresponding author by qualified researchers.
