# Supplementary material for: Long-term outcome of patients presenting with myocardial injury or myocardial infarction
Source: Clin Res Cardiol. 2023 Nov 20;114(6):700–8. doi: 10.1007/s00392-023-02334-w (PMC12089235; doi:10.1007/s00392-023-02334-w)
Supplement: Supplementary file 1 — Supplementary file1 (DOCX 97 KB) [file 392_2023_2334_MOESM1_ESM.docx]

**Supplementary Material**

**Long-term outcome of patients presenting with myocardial injury or myocardial infarction**

Paul M Haller, MD, PhD^1,2^; Caroline Kellner, MSc^1^, Nils A Sörensen, MD^1,2^; Jonas Lehmacher, MD^1^; Betül Toprak, MD^1,2^; Alina Schock^1^, Tau S Hartikainen^,^ MD^3^; Raphael Twerenbold, MD^1,2,4^; Tanja Zeller, PhD^1,2,4^; Dirk Westermann, MD^1,2,3^; Johannes T Neumann, MD, MCR^1,2,5^

1) Department of Cardiology, University Heart and Vascular Center Hamburg, Hamburg, Germany.

2) German Center for Cardiovascular Research (DZHK), Partner Site Hamburg/Kiel/Lübeck, Hamburg, Germany.

3) Department of Cardiology, University Heart Center Freiburg Bad Krotzingen, Bad Krotzingen, Germany

4) University Center of Cardiovascular Science, University Heart and Vascular Center Hamburg, Hamburg, Germany.

5) Department of Epidemiology and Preventive Medicine, School of Public Health and Preventive Medicine, Monash University, Melbourne, Australia.

Table of Content

[Supplementary Table 1: Ambulatory medication at presentation 3](#_Toc145409536)

[Supplementary Table 2: Myocardial stress tests performed in patients with troponin elevation and without type 1 myocardial infarction 4](#_Toc145409537)

[Supplementary Table 3: Adjudicated causes for troponin elevation in patients with acute or chronic myocardial injury 5](#_Toc145409538)

[Supplementary Table 4: Cox-regression model for all-cause mortality 6](#_Toc145409539)

[Supplementary Table 5: Cox-regression model for the composite cardiovascular endpoint 7](#_Toc145409540)

[Supplementary Figure 1: 8](#_Toc145409541)

Supplementary Table 1: Ambulatory medication at presentation

|  | **All (N=2714)** | **STEMI (N=143)** | **NSTEMI 1 (N=236)** | **NSTEMI 2 (N=128)** | **Acute injury (N=86)** | **Chronic injury (N=677)** | **Other (N=1444)** |
| --- | --- | --- | --- | --- | --- | --- | --- |
| Anti-platelet drugs | 1003 (37.4) | 39 (28.5) | 127 (53.8) | 53 (42.1) | 35 (40.7) | 322 (48.4) | 427 (15.7) |
| ACEi/ARB | 1230 (46.0) | 43 (31.2) | 131 (55.5) | 74 (58.7) | 45 (52.3) | 420 (63.6) | 517 (19.0) |
| Beta Blockers | 1059 (39.6) | 34 (24.6) | 107 (45.3) | 63 (50.0) | 35 (40.7) | 399 (60.5) | 421 (15.5) |
| Diuretics | 609 (22.8) | 15 (10.9) | 77 (32.6) | 49 (38.9) | 34 (39.5) | 272 (41.3) | 162 (6.0) |
| Calcium Channel Blocker | 414 (15.5) | 11 (8.0) | 56 (23.7) | 21 (16.7) | 13 (15.1) | 153 (23.3) | 160 (5.9) |
| Statins | 865 (32.3) | 28 (20.1) | 97 (41.1) | 57 (45.2) | 29 (33.7) | 299 (45.4) | 355 (13.1) |
| Antidiabetics | 342 (12.7) | 16 (11.5) | 53 (22.5) | 14 (11.1) | 14 (16.3) | 135 (20.3) | 110 (4.1) |
| Anticoagulation | 434 (16.2) | 10 (7.2) | 24 (10.2) | 36 (28.6) | 25 (29.1) | 204 (30.7) | 135 (5.0) |
| Antianginal drugs | 161 (6.0) | 6 (4.3) | 21 (8.9) | 9 (7.1) | 2 (2.3) | 61 (9.2) | 62 (2.3) |

The ambulatory medication reported at baseline is provided for the overall study population and after stratification by the final diagnosis. The group “others” gathers patients with final diagnoses not involving any elevation of high-sensitivity cardiac troponin T, including stable angina pectoris, unstable angina pectoris, cardiac non-coronary chest pain, and non-cardiac chest pain.

Supplementary Table 2: Myocardial stress tests performed in patients with troponin elevation and without type 1 myocardial infarction

|  | **NSTEMI 2 (N=128)** | **Acute injury (N=86)** | **Chronic injury (N=677)** |
| --- | --- | --- | --- |
| Myocardial stress test performed during or shortly after index presentation, n (%) | 53 (42.1) | 35 (40.7) | 322 (48.4) |
| Positive finding on stress test, n (%) | 1 (0.8) | 0 (0) | 20 (3.0) |

The ambulatory medication reported at baseline is provided for the overall study population and after stratification by the final diagnosis. The group “others” gathers patients with final diagnoses not involving any elevation of high-sensitivity cardiac troponin T, including stable angina pectoris, unstable angina pectoris, cardiac non-coronary chest pain, and non-cardiac chest pain.

Supplementary Table 3: Adjudicated causes for troponin elevation in patients with acute or chronic myocardial injury

|  | **N (%)** |
| --- | --- |
| **Chronic myocardial injury** | **677 (100)** |
| Myocarditis | 6 (0.9) |
| Atrial fibrillation | 59 (8.7) |
| Atrial tachycardia | 24 (3.6) |
| Heart failure | 116 (17.2) |
| Hypertension | 169 (25.0) |
| AV stenosis | 16 (2.4) |
| Bradycardia | 9 (1.3) |
| Pulmonary embolism | 8 (1.2) |
| Aortic disease | 3 (0.4) |
| Pulmonary infection | 6 (0.9) |
| Chronic obstructive pulmonary disease/pulmonary hypertension | 11 (1.6) |
| Non-obstructive coronary artery disease | 93 (13.8) |
| Ventricular tachycardia | 1 (0.1) |
| Anemia | 4 (0.6) |
| Chronic kidney disease | 24 (3.6) |
| Chemotherapy-induced cardiotoxicity | 4 (0.6) |
| Exercise | 1 (0.1) |
| Obstructive coronary artery disease | 95 (14.1) |
| Other | 27 (4.0) |
| **Acute myocardial injury** | **86 (100)** |
| Myocarditis | 9 (10.5) |
| Atrial tachycardia | 2 (2.3) |
| Takotsubo cardiomyopathy | 13 (15.1) |
| Heart failure | 28 (32.6) |
| Hypertension | 3 (3.5) |
| AV stenosis | 5 (5.8) |
| Bradycardia | 1 (1.2) |
| Pulmonary embolism | 12 (14.0) |
| Aortic disease | 3 (3.5) |
| Pulmonary infection | 1 (1.2) |
| Chronic obstructive pulmonary disease/pulmonary hypertension | 1 (1.2) |
| Non-obstructive coronary artery disease | 1 (1.2) |
| Ablation | 1 (1.2) |
| Contusion | 1 (1.2) |
| Sepsis | 2 (2.3) |
| Amyloidosis/sarkoidosis | 1 (1.2) |
| Other | 2 (2.3) |

Supplementary Table 4: Cox-regression model for all-cause mortality

|  | **HR (95% CI)** | **p-value** |
| --- | --- | --- |
| Acute injury | 3.28 (2.09, 5.17) | <0.001 |
| Chronic injury | 2.16 (1.59, 2.93) | <0.001 |
| Non-ST-elevation myocardial infarction Type 1 | 2.15 (1.48, 3.13) | <0.001 |
| Non-ST-elevation myocardial infarction Type 2 | 2.53 (1.66, 3.86) | <0.001 |
| STEMI | 2.31 (1.44, 3.71) | <0.001 |
| Age, years | 1.05 (1.04, 1.06) | <0.001 |
| Male | 1.28 (1.02, 1.59) | 0.029 |
| Diabetes | 1.31 (1.03, 1.67) | 0.029 |
| Ever smoker | 1.41 (1.14, 1.74) | 0.0016 |
| Hypertension | 1.30 (0.98, 1.74) | 0.073 |
| Hyperlipoproteinemia | 0.72 (0.58, 0.90) | 0.0036 |
| Family history of coronary artery disease | 0.63 (0.44, 0.91) | 0.015 |
| Stroke | 1.48 (1.08, 2.02) | 0.015 |
| Congestive heart failure | 1.74 (1.38, 2.19) | <0.001 |
| Chronic kidney disease (eGFR < 60 ml/min) | 1.63 (1.30, 2.04) | <0.001 |
| History of coronary artery disease | 1.25 (1.00, 1.56) | 0.047 |
| N | 2547 |  |
| N events | 398 |  |

Cox-regression model investigating the endpoint all-cause mortality. All patients with diagnoses not involving any troponin elevation (that are stable angina pectoris, unstable angina pectoris, cardiac non-coronary chest pain, non-cardiac chest pain) serve as the reference for the above diagnoses.

Supplementary Table 5: Cox-regression model for the composite cardiovascular endpoint

|  | **HR (95% CI)** | **p-value** |
| --- | --- | --- |
| Acute injury | 1.92 (1.08, 3.43) | 0.027 |
| Chronic injury | 1.59 (1.16, 2.18) | 0.0037 |
| Non-ST-elevation myocardial infarction Type 1 | 2.62 (1.85, 3.69) | <0.001 |
| Non-ST-elevation myocardial infarction Type 2 | 1.33 (0.77, 2.31) | 0.30 |
| STEMI | 3.66 (2.41, 5.57) | <0.001 |
| Age, years | 1.01 (1.00, 1.02) | 0.022 |
| Male | 1.25 (0.97, 1.60) | 0.079 |
| Diabetes | 1.41 (1.09, 1.84) | 0.010 |
| Ever smoker | 1.07 (0.85, 1.35) | 0.54 |
| Hypertension | 1.39 (1.00, 1.93) | 0.051 |
| Hyperlipoproteinemia | 1.13 (0.89, 1.44) | 0.31 |
| Family history of coronary artery disease | 1.07 (0.80, 1.43) | 0.66 |
| Stroke | 1.36 (0.93, 2.00) | 0.12 |
| Congestive heart failure | 1.35 (1.03, 1.78) | 0.030 |
| Chronic kidney disease (eGFR < 60 ml/min) | 1.52 (1.18, 1.96) | 0.0014 |
| History of coronary artery disease | 2.56 (1.97, 3.33) | <0.001 |
| N | 2422 |  |
| N events | 328 |  |

Cox-regression model investigating a cardiovascular composite endpoint (including cardiovascular death, incidental myocardial infraction, or unplanned revascularization). All patients with diagnoses not involving any troponin elevation (that are stable angina pectoris, unstable angina pectoris, cardiac non-coronary chest pain, non-cardiac chest pain) serve as the reference for the above diagnoses.

Supplementary Figure 1:

Flow chart of patient enrolment in the Biomarkers in Acute Cardiac Care cohort study and adjudicated diagnoses according to the Fourth Universal Definition of Myocardial Infarction. NSTEMI – Non-ST-elevation myocardial infarction; STEMI – ST-elevation myocardial infarction
